# Investigating where adolescents engage in moderate to vigorous physical activity and sedentary behaviour: An exploratory study

**DOI:** 10.1371/journal.pone.0276934

**Published:** 2022-12-06

**Authors:** Alex Christensen, Claire Griffiths, Matthew Hobbs, Chris Gorse, Duncan Radley

**Affiliations:** 1 Carnegie School of Sport, Leeds Beckett University, Leeds, United Kingdom; 2 Faculty of Health, University of Canterbury, Christchurch, Canterbury, New Zealand; 3 GeoHealth Laboratory, Geospatial Research Institute, University of Canterbury, Christchurch, Canterbury, New Zealand; 4 School of Built Environment and Engineering, Carnegie, Leeds Beckett University, Leeds, United Kingdom; Guru Ghasidas Vishwavidyalaya: Guru Ghasidas University, INDIA

## Abstract

**Background:**

There is a persistent lack of understanding on the influence of the environment on behaviour and health. While the environment is considered an important modifiable determinant of health behaviour, past research assessing environments often relies on static, researcher-defined buffers of arbitrary distance. This likely leads to misrepresentation of true environmental exposures. This exploratory study aims to compare researcher-defined and self-drawn buffers in reflecting the spaces and time adolescents engage in physical activity (PA) and sedentary behaviour. It also investigates if adolescent’s access the PA facility and greenspace nearest their home or school for PA, as well as examine how much time adolescents spent in PA at any PA facilities and greenspaces.

**Methods:**

Adolescents (aged 14–18 years; n = 34) were recruited from schools in West Yorkshire, England. Seven consecutive days of global positioning system (GPS) and accelerometer data were collected at 15 second intervals. Using ArcGIS, we compared 30 different researcher-defined buffers including: radial, network and ellipse buffers at 400m, 800m, 1000m, 1600m and 3000m and participant-defined self-drawn neighbourhoods to objectively measured PA and sedentary space and PA time. Location of PA was also compared to Points of Interest data to determine if adolescents use the nearest PA facility or greenspace to their home or school and to examine how much PA was undertaken within these locations.

**Results:**

Our exploratory findings show the inadequacy of researcher-defined buffer size in assessing MVPA space or sedentary space. Furthermore, less than 35% of adolescents used the greenspaces or PA facilities nearest to their home or school. Approximately 50% of time spent in PA did not occur within the home, school, PA facility, or greenspace environments.

**Conclusion:**

Our exploratory findings help to begin to quantify the inadequacy of researcher-defined, and self-drawn buffers in capturing adolescent MVPA and sedentary space, as well as time spent in PA. Adolescents often do not use PA facilities and greenspaces nearest their home and school and a large proportion of PA is achieved outside PA facilities and greenspaces. Further research with larger samples are needed to confirm the findings of this exploratory study.

## Introduction

The psychological, sociological and physiological benefits of regular physical activity (PA) for youth are well-known and long established. These benefits, including preventing and managing chronic conditions such as coronary heart disease, stroke, type 2 diabetes, cancer, mental health problems, and musculoskeletal conditions, are achieved when children and adolescents engage in 60 minutes of PA a day [1, 2]. However, many fail to meet this recommendation with over 80% of 13–15 years olds not achieving this guideline globally [3]. Additionally, sedentary behaviour, defined as a cluster of individual behaviours where sitting or lying is the dominant mode of posture [4], is highly prevalent with young people spending up to nine hours of their daily waking time sitting [5]. There is strong evidence that sedentary behaviour affects health independent of PA [6]. Consequently, a clear understanding of factors that influence PA and sedentary behaviour is required.

Local and national strategies increasingly identify the environment as a modifiable determinant of PA and sedentary behaviour [7, 8]. Well-designed neighbourhoods with safe spaces for adolescents to be active, such as cycle lanes have been shown to contribute to increases in PA [9, 10]. Despite this, assessing individual’s environments often relies on static researcher-defined buffers of arbitrary distance (typically between 100–4800 meters) around the home and sometimes school [11, 12]. Recent work highlights that using these measures as exposure to the local residential area lead to a “local” or “residential trap”, which ignores the fact that people live and spatially relate to more than one anchor point [13, 14]. Measuring exposure only at one’s place of residence ignores non-residential locations visited during daily activities, such as the work place and school, and thus may misrepresent true environmental exposures [15]. These methods are criticised as being ‘place-based’ instead of ‘people-based’ [16]. However, it has been shown that adolescents operate beyond a static researcher-defined buffer around the home or school neighbourhood [17–20]. One study in American adolescent females (n = 15) reported participants spent 33% of their awake time more than 1 km from their homes [17]. Another study in adolescents (n = 55) found 90% of their time was spent outside of their home ‘neighbourhood’ [18]. Additionally, the negative health effects of sedentary behaviour, independent of PA, makes identifying factors associated with sedentary behaviour in the environment especially important [21]. For example, it is likely certain features in the environment encourage sedentary behaviour. However, few studies have considered how the environment relates to adolescent sedentary behaviour [22, 23].

Fundamentally, the objective of investigating the built environment’s role in PA behaviour is to define access to PA opportunities so that appropriate public health action can be taken to facilitate and increase PA engagement while decreasing disparities in access [24]. The two-step floating catchment area method attempts to overcome spatial limitations in existing measures of spatial accessibility by modelling real-life access [25]. However, this method is reliant on available empirical data on facilities and its relationship to geography [25]. Catchment areas, defined as the area from which a location, such as a city, service, or institution, attracts a population that uses its services and opportunities, have also been used [26]. Using school or neighbourhood catchment areas, allows for features (e.g., playgrounds, greenspace) within these areas to be accounted for and tested for associations with PA levels. Catchment area methods are constrained however, due to being based on arbitrary features to define the area.

Proximity metrics, which measure the distance between the home and one or more location, are a commonly used method [27]. Past research theorises that proximity of PA facilities and greenspace to the home will have a positive association with PA levels [28–30]. However, research supporting this theory has had mixed results [28, 31, 32], possibly due to several assumptions. First, past research has assumed that the effect of distance on behaviour is linear (i.e. each increment in distance results in a decline in the likelihood of a behaviour occurring). Second, that individuals are aware of all the available PA opportunities. Third, that choice is guided by distance. Finally, much of this research is based the presence of a PA opportunity, rather than information on whether as opportunity is actually used [31]. These limitations are largely due to a lack of data on actual use and lack of knowledge about the precise spatial and temporal configuration of the factors, both physical and social, that exert an influence on individual behaviour [16]. Current research suggests that individualised measures of the environment, such as using GPS, could lead to different and a more accurate understanding of environmental exposure [15, 33].

While it is recognised that a move beyond static concepts and methods of conventional notions of geographic context and exposure measures is required [16, 20] few studies have quantified how adolescents engage with PA facilities or how accurately researcher-defined buffers reflect the time adolescents engage in PA. Therefore, this exploratory study responds to calls to better understand where adolescents are active [34]. The research questions are:

1. Do researcher-defined buffers reflect the spaces that adolescent engage in activity behaviour?;
2. Do self-drawn neighbourhoods reflect the spaces that adolescents engage in activity behaviour?
3. Do adolescents access the closest PA facility or park to their home or school for PA?;
4. Do adolescents use PA facilities or parks to be physically activity?;
5. Do researcher-defined buffers capture the time adolescents engage in PA behaviour?

## Methods

### Participants and setting

Adolescents aged 14–18 years were recruited from two secondary schools in West Yorkshire, England. Recruitment took place between May 2017 and March 2018. Sixty-nine participants (24 male, 45 female) provided written parental consent and written participant assent to participate in the study. Data collection occurred in two waves, autumn (September/October 2017) and spring (March/April 2018), due to restrictions in school timetables. Individual demographics data on age, gender, postcode, and ethnicity (amalgamated into White British and all other ethnic groups due to small sample sizes in other ethnic groups) were collected using an online questionnaire developed in Qualtrics (Qualtrics, Provo, USA) (Qualtrics questionnaire is available in S1 File). Institutional approval was received from Carnegie School of Sport, Leeds Beckett Research, Research Ethics Committee (ref: 37750).

### Daily movement

To objectively collect individual’s daily movement, participants either wore a GPS device (Garmin Forerunner 401) (n = 39) or ran a proprietary GPS smartphone application (Tracker) (n = 30) for seven consecutive days, collecting data over 15 second epochs [35]. Providing two options of GPS was used as an attempt to increase compliance as 96% of young people (16–24 years old) use a smartphone [36]. Additionally, smartphone GPS has been found to be accurate within 100 metres [37, 38]. Participants were instructed to wear the device during all waking hours, expect if they were participating in a water-based activity such as swimming. GPS data were visually inspected and cleaned to ensure that any data outside of the study period was removed and data were separated out by days, using time stamps, and total daily wear time was calculated.

The ActiGraph GT3 series (GT3x+, GT3x, and WGT3x+), and GT9X (ActiGraph, Pensacola, FL) were used to objectively measure PA and sedentary behaviour. Different models were used due to availability, however research supports the compatibility of these models, with high correlations between the X, Y, and Z axes [39] and high intraclass correlation coefficients [40]. Data were collected over 15 second epochs. Accelerometer cut-points were applied to accelerometer data [41, 42]. These cut-points provided three levels of activity: sedentary (0–25 counts per 15 second epoch (CPE)), light (26–573 CPE), moderate to vigorous (MVPA) (≥574–1002 CPE).

GPS and accelerometer data were combined by matching date and timestamps in Excel (version 365; Microsoft Corporation, Redmond, WA, USA), resulting in each GPS point having a corresponding count of PA. Combined data was transferred to ArcGIS (v. 10.6.1) for analysis. Valid day criteria was set to ≥5 hours a day [43] and data determined to be in MVPA (>574 CPE) was separated into its own feature layer. GPS have been found to exhibit average errors up to 100 metres but can vary by the model and age of the device[37, 38]. It was seen as important to account for GPS inaccuracy when creating individual activity space therefore, a 100 metre buffer was created around MVPA points to create MVPA space (km^2^). Similarly, data determined to be at sedentary behaviour (≤25 CPE) was also separated into its own feature layer and 100 m buffer was created around the sedentary data to create sedentary space. MVPA space and sedentary space size was determined by calculating the area the 100 metre buffer covered and was reported in square kilometres (km²) [44].

### Researcher- and participant defined neighbourhoods

#### Radial, network and ellipse buffers

Within ArcGIS (v. 10.6.1), 30 types of buffers were created, centred on the home (based on postcode) and school of each participant. Radial, street network buffers, straight line ellipse (SLE) and network line ellipse (NLE) were created at five buffer sizes typically used in the research (400m, 800m, 1km, 1600 km and 3km). Straight line and road network paths (based on the shortest network route) between the home and school were used to create ellipse buffers (described in detail in Christensen et al. (2020) [20])

#### Self-drawn neighbourhoods

Participants were given instructions to complete a self-drawn neighbourhood (SDN) activity within Google My Maps. Participants entered their postcode and were asked to “*create a boundary of what you consider as your neighbourhood on the map*”. This was left open for interpretation for the participant as to what “neighbourhood” meant to them in order to provide a richer understanding of what residents perceive a neighbourhood to be. Participants’ SDN’s were downloaded from Google My Maps and converted to a feature in ArcMap, allowing for visualisation and comparison. SDN size were than calculated by determining the area the SDN covered in square kilometres (km²). Examples of self-drawn neighbourhoods can be found in S1 Fig.

#### Location of physical activity facilities and greenspace

Using the Points of Interest (POI) Classification Scheme [45], a database as previously defined by [46] was amalgamated to represent PA facilities and imported into ArcGIS. A full list of facilities included can be found in S1 Table. The X, Y location of each POI was mapped in ArcGIS and projected using the British National Grid coordinate system. Duplicates were removed by dissolving the feature and those with the same coordinates aggregated. Additionally, public parks and allotments were added from Ordnance Survey Open Greenspace data [47].

### Analysis

**Fig 1:**
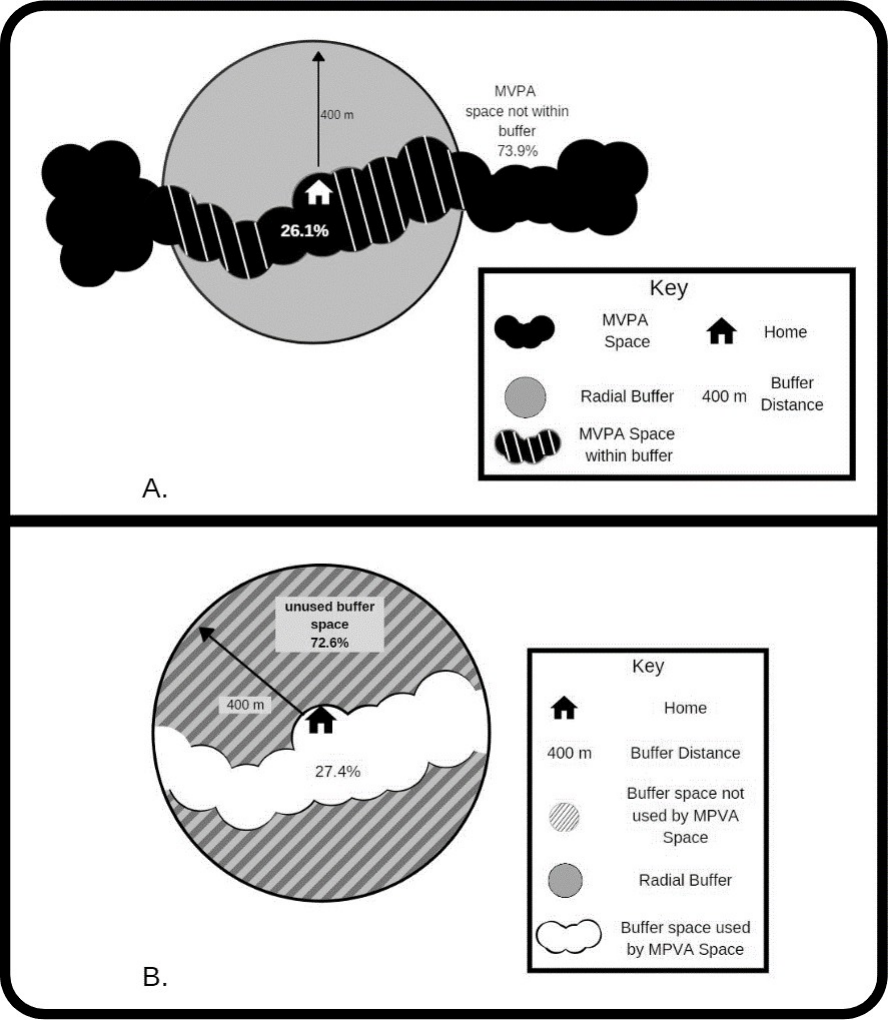
Panel A) A visual example depicting 26.1% of MVPA space within 400 m home radial buffer compared to 73.9% MVPA space occurring outside of buffer radius. **Panel B)** A visual example depicting 27.5% of 400 m home radial buffer used by MVPA space, 72.6% of buffer is not used by MPVA space.

All researcher-defined buffers were assessed to determine how accurately they captured MVPA space and sedentary space. Within ArcGIS (v. 10.6.1), the area and percentage of intersecting features (e.g. the percentage of space within the buffer) was calculated. The percentage of space within all buffer types and sizes, were calculated (Fig 1, Panel A). Additionally, the percentage of the unused buffer space was determined to define how much of the buffer misrepresented space (Fig 1, Panel B). Finally, the percentage of MVPA and sedentary space within self-drawn neighbourhoods and the amount of unused self-drawn neighbourhood space were determined.

To determine if PA facilities or greenspace were used for PA, first a 100-metre buffer was created around all PA facilities and greenspace within MVPA space. The count of combined GPS and accelerometer points within MVPA was then determined for both PA facilities and greenspace independently, and the total time in MVPA at each location determined. Additionally, the percentage of total MVPA time at each location was calculated. The same process was followed to determine how many MVPA minutes and total MVPA time were spent at home and school.

To determine if adolescents access PA facilities and greenspace nearest to their home or school for MVPA, the closest PA facility and greenspace to each participants’ home and school location was determined, based on Euclidean distance within ArcGIS. If the facility or greenspace was within or intersected with the MVPA space boundary, it was determined to be accessed by the participant.

To investigate how accurately researcher-defined buffers reflect the time adolescents engage in MVPA behaviour, the amount of MVPA time that was spent within each buffer was calculated. MVPA time was used rather than MVPA space in order to weight results by time spent with buffer, rather than if the space was accessed or not. The average percent of MVPA time captured by the various buffer types and sizes was then determined.

## Results

### Participant characteristics

Study sample characteristics are described in Table 1. This study had a relatively high participant burden and asked participants to complete multiple tasks and comply over a weeklong time period, resulting in only 34 participants providing valid combined GPS and accelerometer data. This loss to follow-up is due to low compliance (e.g. not turning GPS device on or wearing the accelerometer). Those who dropped out had minimal differences to included participants (S2 Table). Overall, there were more female and White British participants, and more participants in most deprived areas.

**Table 1:** Study sample characteristics (n = 34).

Characteristic	Frequency
**Gender**	
Female	24 (70.6)
Male	10 (29.4)
**Age** ^**1**^	16.2 ± 1.2
**Ethnicity**	
White British	27 (79.4)
All other ethnic backgrounds	7 (20.6)
***Area Level Deprivation***	
1 (most deprived)	11 (32.4)
2	7 (20.6)
3	4 (11.8)
4	7 (20.6)
5 (least deprived)	5 (14.7)
**Percent of Total Time Spent in MVPA** ^1^	8.43 ± 4.15
**Percent of Total Time Spent Sedentary** ^1^	71.01 ± 4.83
**Self-drawn Neighbourhood Size** (km^2^) (n = 53)^1^	0.62 ± 1.1
**MVPA Space Size** (km^2^) (n = 34)^1^	0.62 ± 0.46
**Sedentary Space Size** (km²) (n = 34)^1^	2.2 ± 2.3

Data are presented as n (%) unless stated otherwise.

^1^Mean ± SD.

Area level deprivation is defined as an area’s potential for health risk; areas with higher deprivation may be more disadvantaged in regard to other area characteristics that influence health.

### RQ1: Do researcher-defined buffers reflect the spaces that adolescent engage in activity behaviour?

#### Captured MVPA space and sedentary space

The percentage of MVPA and sedentary space captured within the radial, network and ellipse buffers around the home and school are presented in Table 2. Network buffers, both home and school based, captured the least amount of MPVA and sedentary space. Straight line and network line ellipse buffers captured notably more MVPA and sedentary space than other buffer types.

**Table 2:** Percentage of MVPA and sedentary space within six buffers by five buffer distances.

	Buffer Type					
Buffer Distance	Home (Radial)	Home (Network)	School (Radial)	School (Network)	Ellipse (Straight Line)	Ellipse (Network Line)
**MVPA**						
400 m	26.1 (8.8, 59.0)	13.5 (1.9, 38.2)	31.0 (7.1, 73.0)	9.1 (1.3, 22.8)	68.3 (18.8, 100)	70.4 (18.3, 100)
800 m	39.5 (13.5, 94.7)	27.8 (7.8, 74.1)	45.2 (7.8, 98.6)	27.8 (5.1, 61.3)	83.0 (25.1, 100)	83.0 (25.2, 100)
1 km	45.4 (13.5, 100)	33.1 (11.5, 85.3)	48.8 (8.8, 100)	37.5 (7.6, 78.1)	85.4 (29.1, 100)	85.5 (29.2, 100)
1.6 km	56.8 (14.7, 100)	44.3 (13.5, 88.0)	57.3 (8.8, 100)	49.4 (8.8, 100)	86.6 (32.15, 00)	90.3 (32.2, 100)
3 km	74.2 (18.9, 100)	61.3 (15.3, 100)	68.4 (8.8, 100)	61.5 (8.8, 100)	92.8 (32.2, 100)	93.5 (38.6, 100)
**Sedentary**						
400 m	14.6 (2.1, 53.1)	6.5 (0.2, 32.5)	16.3 (2.0, 70.1)	4.8 (0.5, 16.4)	43.4 (8.6, 100)	52.2 (9.0, 99.3)
800 m	24.0 (5.9, 78.0)	14.7 (1.6, 51.1)	26.7 (2.8, 95.4)	15.0 (2.1, 49.5)	61.7 (19.3, 100)	64.8 (19.5, 100)
1 km	29.9 (8.0, 100)	17.7 (5.6, 52.3)	32.4 (3.4, 100)	20.4 (2.8, 70.2)	67.6 (24.7, 100)	69.2 (27.4, 100)
1.6 km	43.8 (11.9, 100)	29.1 (10.1, 93.5)	43.9 (6.7, 100)	33.4 (4.5, 100)	79.1 (40.4, 100)	79.2 (44.3, 100)
3 km	69.9 (20.9, 100)	55.5 (18.9, 100)	67.7 (12.2, 100)	53.1 (9.5, 100)	90.1 (57.8. 100)	90.5 (57.8, 100)

Mean value (minimum value, maximum value).

#### Unused buffer space

The percentage of the researcher-defined buffer that was unused by MVPA space and sedentary space is shown in Table 3. This is explained visually in Fig 1B. All types of 400m buffers had the least amount of unused space, while 3 km buffers had the greatest amount of unused space. Home and school network buffers, when compared to the corresponding type of buffer at equivalent buffers distances, had the least amount of unused space at all distances. All buffers demonstrated a high amount of unused space with the least amount of unused space being 39.4% (sedentary 400m home network), and with all 3km buffer types having more than 93% of buffer space unused.

**Table 3:** Percentage of buffer unused by MVPA and sedentary space.

	Buffer Type					
Buffer Distance	Home (Radial)	Home (Network)	School (Radial)	School (Network)	Ellipse (Straight Line)	Ellipse (Network Line)
**MVPA**						
400 m	72.6 (28.3, 93.1)	45.7 (0.3, 83.4)	70.7 (45.1, 85.7)	63.1 (22.6, 98.3)	84.5 (53.1, 97.42)	86.9 (61.7, 97.7)
800m	88.6 (63.1, 98.3)	73.6 (40.8, 96.1)	88.9 (68.7, 96.1)	77.9 (55.3, 88.3)	92.1 (76.6, 98.8)	93.2 (80.7, 98.9)
1 km	91.3 (68.7, 98.9)	79.9 (42.5, 97.6)	92.1 (76.0, 97.5)	82.4 (64.8, 92.0)	94.1 (81.6, 99.1)	94.8 (85.7, 99.2)
1.6 km	95.7 (84.8, 99.6)	90.6 (64.8, 99.1)	96.2 (89.1, 99.0)	92.2 (81.4, 97.5)	96.9 (91.5, 99.5)	97.2 (92.2, 99.5)
3 km	98.4 (94.9, 99.7)	96.8 (88.4, 99.8)	98.7 (96.0, 99.7)	97.4 (92.2, 99.4)	98.80 (96.6, 99.8)	98.9 (96.7, 99.8)
**Sedentary**						
400 m	64.8 (30.1, 90.6)	39.4 (5.0, 99.4)	63.0 (31.4, 93.8)	46.0 (2.5, 96.7)	79.4 (56.4, 94.9)	79.6 (58.5, 95.3)
800m	83.8 (67.0, 97.7)	66.8 (26.5, 98.9)	84.5 (67.6, 98.4)	67.5 (26.3, 96.5)	86.8 (72.9, 97.9)	88.1 (77.7, 98.0)
1 km	86.9 (71.1, 98.5)	74.4 (46.8, 97.0)	87.7 (70.8, 99.0)	74.4 (44.7, 97.4)	89.1 (79.5, 98.5)	90.2 (81.2, 98.6)
1.6 km	92.1 (82.3, 98.6)	85.7 (63.0, 94.9)	93.1 (82.3, 99.0)	86.9 (69.9, 98.1)	92.9 (85.4, 98.9)	93.5 (87.2, 99.0)
3 km	96.1 (91.6, 99.5)	93.3 (83.6, 99.0)	95.4 (61.8, 99.3)	94.2 (80.9, 99.0)	96.6 (91.1, 99.5)	96.8 (91.5, 99.5)

Mean value (minimum value, maximum value).

#### Comparison of buffers

**Fig 2:**
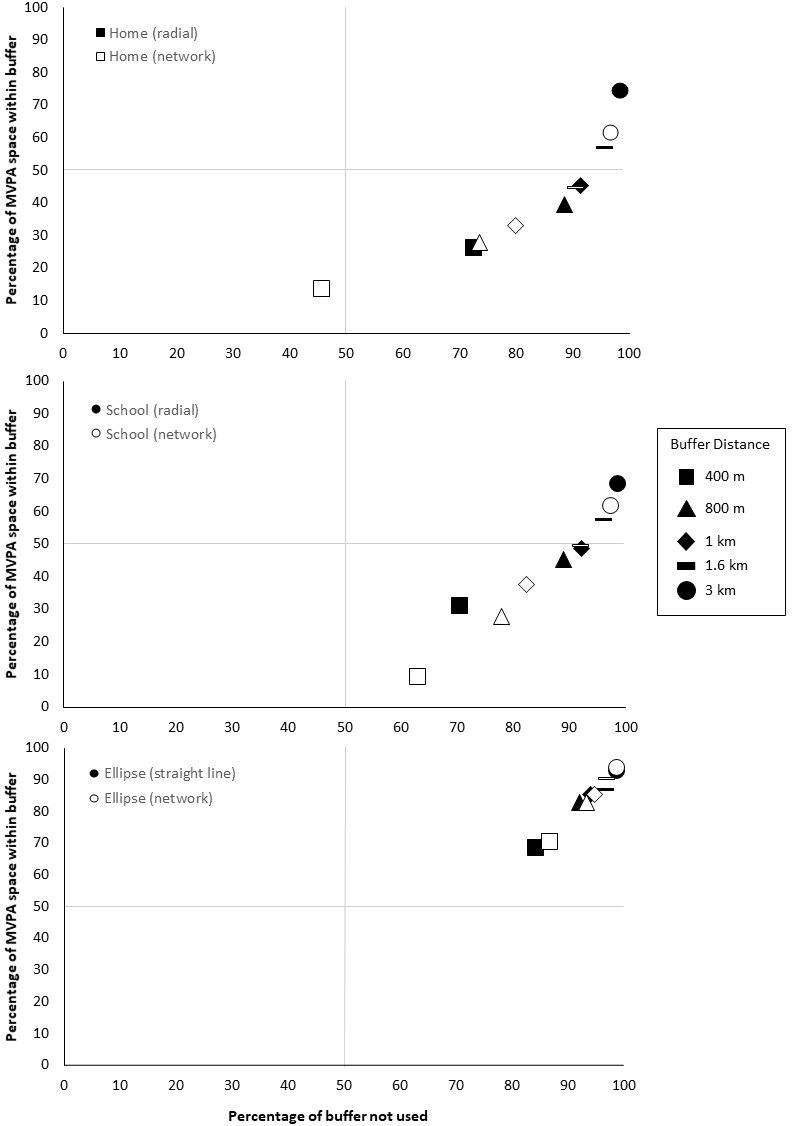
Comparison of buffers in capturing MVPA space and unused space by home, school and ellipse.

**Fig 3:**
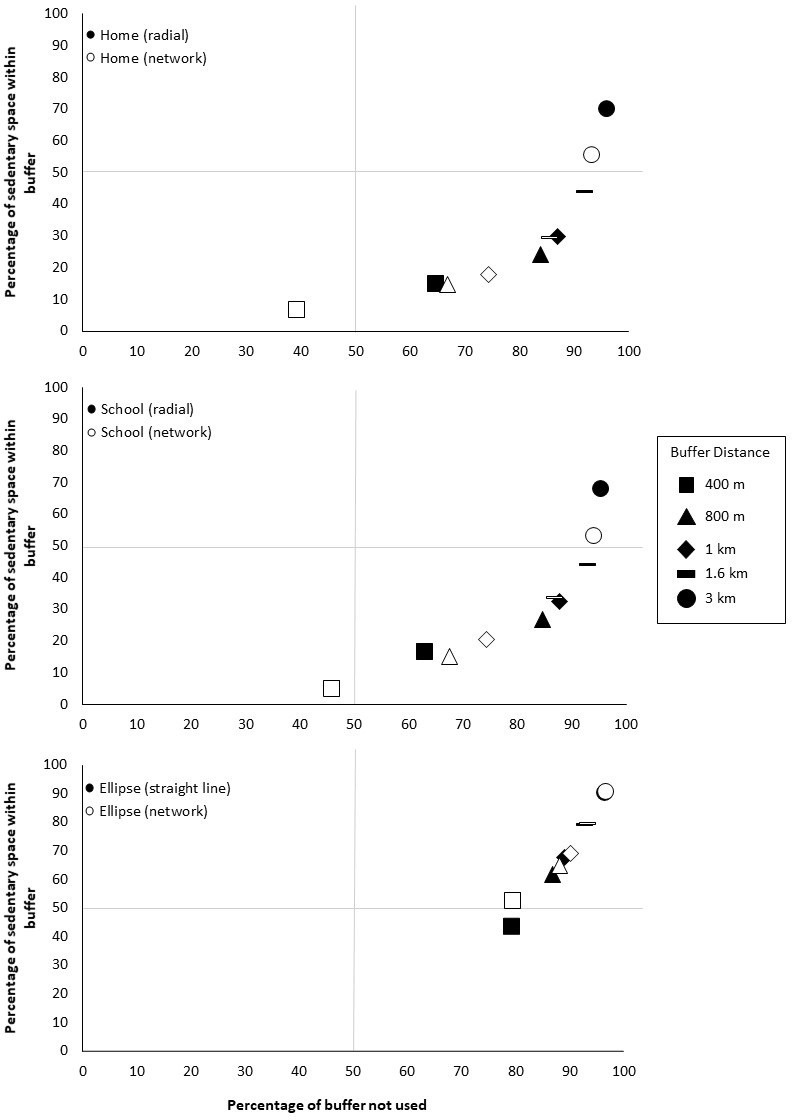
Comparison of buffers in capturing sedentary space and unused space by home, school and ellipse.

To compare each researcher-defined buffer, results from within buffer and unused buffer space were plotted to allow visualisation of how accurate each buffer was for capturing MVPA space and sedentary space. Fig 2 shows a scatterplot of each buffer type and buffer size by how much MPVA was within the buffer and how much unused buffer space was captured, where Fig 3 shows the same for sedentary space.

For both MVPA space and sedentary space, as buffer size increases the amount of space within increases and the amount of unused space also increases. No buffer falls within the upper left quadrant with a low amount of unused space or high amount of actual use captured. All ellipse buffers tended to capture the most MVPA and sedentary space, but also had the highest amount of unused space. Home 400m network buffers for MVPA space and home and school 400m network buffers for sedentary space, were the only buffer within the lower left quadrant, meaning they had low amounts of activity space within the buffer and low amounts of unused buffer space. All other buffers fell within the lower right quadrant, with moderate to high amounts of MVPA or sedentary space within the buffer and moderate to high amounts of unused buffer space. Overall, when considering both space within buffers and unused buffer space, no buffer was the ideal at capturing MVPA or sedentary space.

### RQ2: Do self-drawn neighbourhoods reflect the spaces that adolescent engage in activity behaviour?

Twenty-eight participants provided self-drawn neighbourhoods, MVPA space, and sedentary space measures. The amount of MVPA space and sedentary space within self-drawn neighbourhoods was calculated (Table 4). Additionally, the amount of unused SDN by MVPA and sedentary space was calculated. On average, 17.6% of MVPA space occurred within SDN and 10.8% of sedentary space occurred within SDN. Furthermore, 54.1% of self-drawn neighbourhoods were used by MVPA space, and 45.0% of SDNs were accessed by sedentary space. This suggests 45% of SDNs had no sedentary or MVPA behaviours.

**Table 4:** Amount of space used between self-drawn neighbourhoods and MVPA space and sedentary space.

SDN unused by MPVA	MVPA within SDN	SDN unused by sedentary space	Sedentary space within SDN
54.1	17.6	45.0	10.8
(0, 98.0)	(1.2, 69.2)	(0, 92.1)	(0.8, 44.1)

Mean value (minimum value, maximum value).

### RQ3: Do adolescents access the closest PA facility or park to their home or school for PA?

Twelve participants (35%) accessed the closest PA facility and 12 (35%) accessed the closest greenspace based on their home location in the seven days of data collection. Based on the school location, only six participants (18%) accessed the closest PA facility and three participants (9%) accessed the closest greenspace.

### RQ4: Do adolescents use PA facilities or parks to be physically activity?

**Fig 4:**
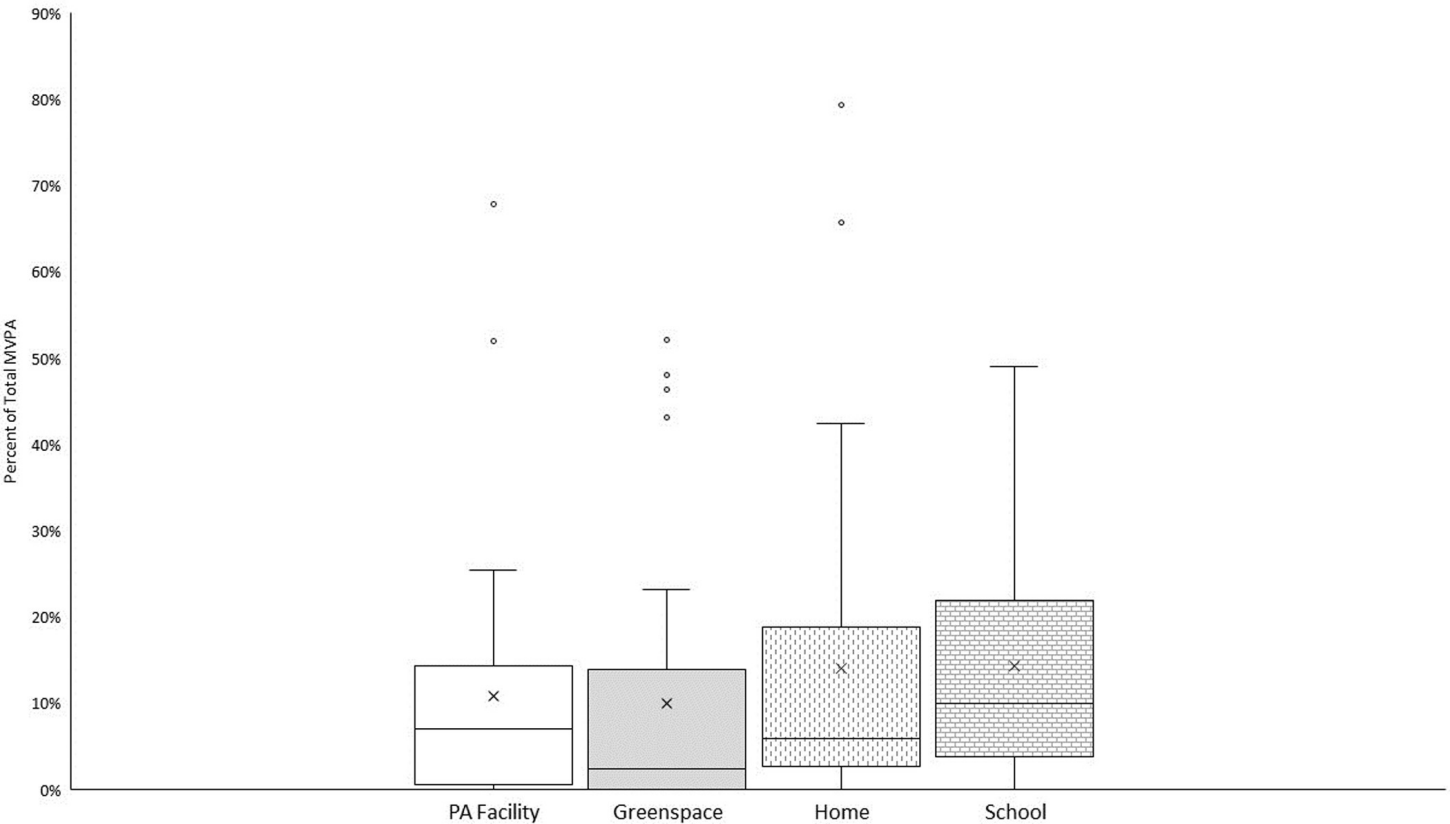
Box plot of percentage of total time spent in MVPA at each location. X represents the mean.

An average of nine and ten minutes were spent in MVPA at home and school locations, respectively. Of the total amount of time spent in MVPA, the home and school locations contributed to 28% of total weekly MVPA time (14% each). An average of ten minutes was spent in MVPA at PA facilities and an average of nine minutes was spent in MVPA in greenspace. Of the total amount of time spent in MVPA, 11% of MVPA time occurred at PA facilities, while 10% of MVPA time occurred in greenspace. Results indicate that on average greater than 50% of time spent in MVPA did not occur at the home, school, within a PA facility or within greenspace. However, the percent of total MVPA time spent at each location varied considerably among participants (see Fig 4).

### RQ5: How accurately do researcher-defined buffers capture the time adolescent engage in PA behaviour?

Table 5 reports the average MPVA time captured by the researcher-defined buffers. Results demonstrate considerable differences in the amount of MVPA captured. Independently, home and school buffers at best, captured 77% of MVPA time (3 km home radial) and at worst 8% (400 metre school network). Home and school network buffers captured notably less MVPA time compared to radial buffers of equivalent distances. For example, at a 400 metre distance, network buffers encompassed 8–20% of MVPA time, while radial buffers captured 32–39%. Ellipse buffers encompassed notably more MVPA than home and school network and radial buffers, at all distances, with 3 kilometre ellipse buffers capturing 94% of MVPA time. Straight line ellipse buffers and NLE buffers were relatively similar at equivalent buffer distances. For example, at 400 metres, a SLE captured 77.5% of MVPA time while an NLE captured 79.7% of MVPA time.

**Table 5:** The percentage of MVPA time undertaken within researcher-defined buffers (n = 34).

	Buffer Type					
Buffer Distance	Home (Radial)	Home (Network)	School (Radial)	School (Network)	Ellipse (Straight Line)	Ellipse (Network Line)
400 m	39.1 (7.8, 87.1)	20.1 (0, 87.1)	32.1 (5.9, 86.1)	8.0 (0.5, 24.5)	77.5 (28.9, 100)	79.7 (27.2, 100)
800 m	48.6 (7.8, 100)	40.4 (4.6, 99.1)	39.7 (6.0, 100)	28.7 (6.0, 67.8)	87.0 (36.1, 100)	86.8 (36.1, 100)
1 km	52.9 (7.8, 100)	43.2 (6.7, 99.1)	45.2 (9.0, 100)	38.3 (9.0, 99.1)	88.7 (38.6, 100)	88.6 (43.5, 100)
1.6 km (1 mile)	61.2 (7.8, 100)	50.9 (7.8, 100)	54.8 (9.0, 100)	44.8 (9.0, 100)	91.4 (45.8, 100)	92.5 (48.9, 100)
3 km	77.4 (18.7, 100)	63.6 (7.8, 100)	66.1 (9.0, 100)	59.4 (9.0, 100)	94.3 (45.9, 100)	94.6 (50.4. 100)

Mean value (minimum value, maximum value).

## Discussion

This exploratory study responds to calls to better understand where adolescents are active [34]. We demonstrate the inadequacy of researcher-defined and self-drawn buffers in reflecting the spaces adolescents engage in moderate to vigorous physical activity (MVPA) and sedentary behaviour. We also found that few adolescent access the PA facilities, such as leisure centres and greenspace, nearest to their home or school for MVPA. Additionally, little overall time is spent in MVPA at any PA facilities and greenspaces. Lastly, we demonstrate a considerable difference in the amount of MVPA time captured by different researcher-defined buffers.

This exploratory study demonstrates the inadequacy of researcher-defined buffers in measuring adolescent activity behaviour. It builds upon the current evidence base by measuring both space within and unused space to compare buffers. This allows for a full picture of how buffers represent an adolescents’ behaviour [20]. When space captured and unused space are assessed together, a high level of unsuitability was shown among all buffer sizes and types. Previous research suggests this is due to the neighbourhood effect averaging problem. This refers to the problem that individual mobility-based exposures to environmental factors tend towards the mean level of the participants or population of a study area when compared to their residence-based exposures [48], ignoring people’s daily mobility and exposures to non-residential contexts and may lead to erroneous results. While the results from this exploratory study are expected, this is the first time these assumptions have been quantified across multiple buffer types and distances in assessing MVPA and sedentary space in adolescents. Overall, the findings from this study, continue to highlight a tension between balancing the capture of behaviour and reducing the amount of unused space. Without more dynamic definitions, the impact of the environment on behaviour will continue to be underestimated [48].

Additionally, when considering activity behaviours, there is also a mismatch between what they identify/perceive as their neighbourhood boundary and behaviours. Only 18% of MVPA space and only 11% of sedentary space were within SDNs. Similar results have been found in previous research [18, 20, 49]. For example, Christensen et al. [20], found approximately 40% of perceived environmental neighbourhoods aren’t even accessed when compared to objectively measured daily movement. These findings suggest that PA or sedentary behaviour may not drive how individuals define their neighbourhood. This highlights the importance of understanding what defines an individual’s neighbourhood or what behaviours influence this definition in order for policies and changes to the environment to have wide-scale impact. This will impact future planning as it typically focuses on developing the neighbourhood environment to promote healthy lifestyles and reduce sedentary behaviour (as seen with the six elements of creating a healthy community [50]). While it is not to say that continual neighbourhood improvement is unimportant, the findings from this paper highlight that other areas beyond just the home environment (e.g. school locations or greenspaces) may be as important to individuals.

Findings showed that only 35% of adolescents used PA facilities or greenspaces closest to their home, and this dropped to 18% and 9% when examining PA facility or greenspace use respectively, nearest to their school. Past research has theorised that proximity of PA facilities and greenspace to the home have a positive association with PA levels [28–30] however research supporting this theory in adults has had mixed results [28–31]. It is likely that the conflicting results are, in part, due to the limitations of the methods used. Studies rely on the presence of a PA opportunity as a proxy for exposure [28–31, 51] which has significant limitations as it assumes individuals are aware of all the available PA opportunities and that choice is guided by distance, as well as assuming that if a PA opportunity is present, it will be used for MVPA [31].

Findings in the present study indicated that approximately 50% of time spent in MVPA did not occur within one of the four investigated environments; 14.1%, 14.3%, 10.8% and 10.0% of MVPA occurred in the home, school, PA facility or greenspace environment, respectively. This reflects previous research, within a large sample of adolescents (n = 1,304; 12–15 year olds), where only 27% of participants reported using their closest park for PA [52]. Furthermore, in a study of 12–16 year olds, data captured using GPS and accelerometers found that only 30% of MVPA time occurred in the home and school environments [53]. Combined with the results from this study, findings demonstrate that adolescents use locations other than the home, school, PA facilities and greenspaces for a large proportion of their MPVA. This calls to question the longstanding assumption within the field that proximity matters, when in reality, spatial choice and spatial interactions need to be more carefully considered [31]. The need to consider where adolescents actually participate in PA and what drives their behaviour is also important to consider moving forward, in order to begin to unpick what influences PA. These results suggest that simply building more PA facilities or greenspaces may not increase PA in this age group. Additionally, understanding the key influences that drive sedentary behaviour and how to reduce the proportion of sedentary time is important. Consideration needs to be given to how other underlying factors, such as perceptions of safety or social groups, influence where individuals choose to be physically active. Furthermore, targeting transport policies as well as consideration on how to replace sedentary behaviours, such as travelling by car, with more active modes is necessary. This provides support that it may be more important for future planning practices to create meaningful places and spaces (i.e. where individuals feel connected) to individuals which potentially have a greater influence on promoting healthy lifestyles. Future research should seek to understand where adolescents spend the other 50% of their time being physically active.

While radial and network buffers poorly reflected time adolescents engage in MVPA and sedentary behaviour (5–74%), results indicate that when accounting for both the home and school by using ellipse buffers, notably more behaviour was captured. This finding suggests a potentially better method of capturing behaviour by creating static spatial units that involves more than one key location (i.e. school or work). This finding may hold promise for future research using secondary data or working with large samples, when collecting GPS data is unrealistic. Past research supports this assertion, indicating that this is due to missing a large proportion of daily time spent outside the home location [54, 55]. This is reinforced by current arguments within the field that traditional methods ignore the understanding of human mobility and are too “place-based” instead of “people-based” [16]. Furthermore, by continuing to only focus on a sole location, researchers fall into the “residential trap” by ignoring non-residential locations and ignoring the fact that people live and spatially relate to multiple points, and thus misrepresent true environmental exposure [13–15]. Researchers need to be aware of, and acknowledge, the significant limitations of only using the home location. Future research should consider accounting for multiple exposures, such as home and school or home and workplace, as this may have potential to improve the amount of actual behaviour captured and allow a better understanding of the relationship between environments and health outcomes.

Several factors limit the results presented within this exploratory study and all results should be interpreted with these study limitations in mind. First, is the small sample size, meaning the findings within this paper are not generalisable. Second, neighbourhood preference and self-selection were not accounted for in the analysis. While adolescents have more independent mobility than children and thus have more control of areas they spend time, parental self-selection of home neighbourhood and/or school could be important to account for in future analyses [56–58]. In addition, a recent study [59] found that 12 days of GPS and accelerometer monitoring would reliably estimate minutes in both low and medium threshold MVPA for many important built environment locations. This suggests the current research’s valid day criteria may be inadequate to accurately assess activity behaviour. Future research should consider longer monitoring timeframes to ensure the data is reliable., There may also be moderating effects we could not detect due to the small sample. For instance, past research [60, 61], has suggested that individuals of higher social-economic status are more likely to reside in more favourable environments (e.g. more available PA opportunities). Lastly, although the perceptions of participants were investigated through drawing of their SDNs; it would have been beneficial to also draw them in relation to PA to better understand the perceived locations of PA. Future research should seek to understand different definitions of perceived neighbourhoods.

## Conclusion

In summary, our exploratory study highlights the inadequacy of using researcher-defined and participant defined buffers to reflect adolescents MVPA and sedentary space. We also found that the majority of adolescents do not access the PA facility or greenspace nearest to their home or school. Additionally, a large proportion of MVPA occurs outside of PA facilities, greenspaces, home or school environments. We also demonstrate a considerable difference in the amount of MVPA time captured by different researcher-defined buffers. This has significant implications for interpreting past research and planning future studies. Future research is needed to confirm our exploratory findings. Additional evidence may wish to investigate what influences the location of adolescent PA in order to find potential leverage points to increase PA.

## Supporting information

S1 Fig Examples of participants self-drawn neighbourhoods. Blue pin represents home; white building represents school. Images are at a 1cm = 1.5km scale.
(TIFF)

S1 Table The groups, categories, and classes of physical activity facilities from Points of Interest Classification Scheme. (DOCX)

S2 Table Participant characteristic by full sample and included participants. (DOCX)

S1 File Qualtrics research questionnaire. (DOCX)
