# Supplementary figures and images for: Investigating where adolescents engage in moderate to vigorous physical activity and sedentary behaviour: An exploratory study
Source: PLoS One. 2022 Dec 6;17(12):e0276934. doi: 10.1371/journal.pone.0276934 (PMC9725162; doi:10.1371/journal.pone.0276934)

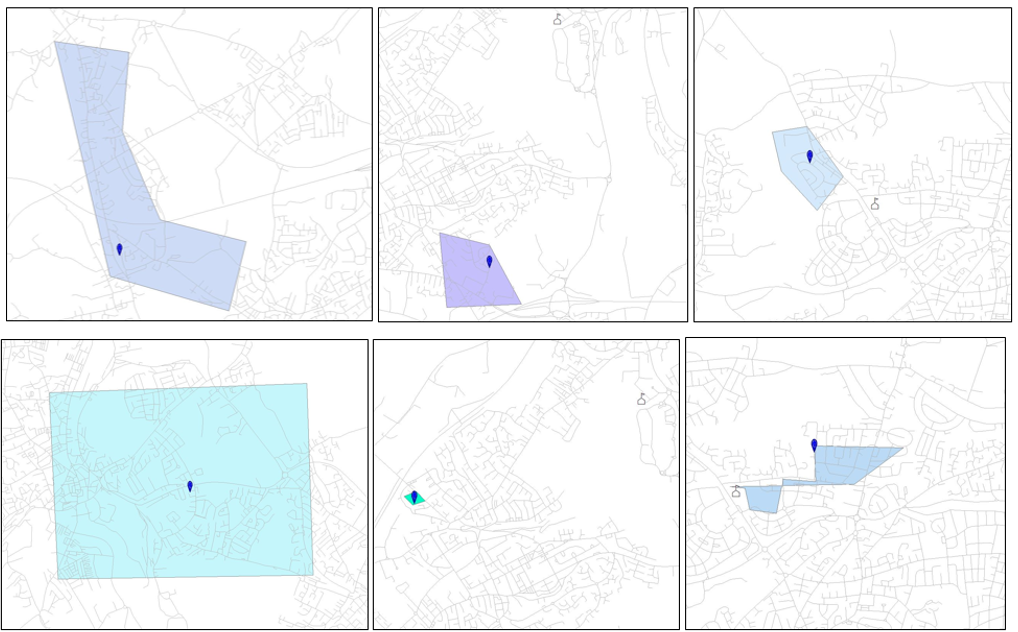

Supplement: S1 Fig — Blue pin represents home; white building represents school. Images are at a 1cm = 1.5km scale. (TIFF) [file pone.0276934.s001.tiff]
