# Supplementary material for: Investigating where adolescents engage in moderate to vigorous physical activity and sedentary behaviour: An exploratory study
Source: PLoS One. 2022 Dec 6;17(12):e0276934. doi: 10.1371/journal.pone.0276934 (PMC9725162; doi:10.1371/journal.pone.0276934)
Supplement: S1 Table — (DOCX) [file pone.0276934.s002.docx]

| **Group** | **Category** | **Class** | **Description** |
| --- | --- | --- | --- |
| 03 | 18 | 0255 | Playgrounds |
| 04 | 23 | 0321 | Riding schools, livery stables and equestrian centres |
| 04 | 23 | 2087 | Water sports |
| 04 | 24 | 0289 | Athletics facilities |
| 04 | 24 | 0290 | Bowling facilities |
| 04 | 24 | 0291 | Climbing facilities |
| 04 | 24 | 0292 | Golf ranges, courses, clubs and professionals |
| 04 | 24 | 0293 | Gymnasiums, sports halls and leisure centres |
| 04 | 24 | 0294 | Ice rinks |
| 04 | 24 | 0300 | Ski infrastructure and aerial cableways |
| 04 | 24 | 0302 | Sports grounds, stadia and pitches |
| 04 | 24 | 0303 | Squash courts |
| 04 | 24 | 0304 | Swimming pools |
| 04 | 24 | 0305 | Tennis facilities |
| 04 | 24 | 0306 | Velodromes |
| 05 | 32 | 0384 | Ballet and dance schools |
| 05 | 32 | 0395 | Martial arts instruction |
| 05 | 32 | 0399 | Sailing schools |
| 05 | 32 | 0400 | Sports and fitness coaching |

Supplementary Table 2. The groups, categories, and classes of physical activity facilities from Points of Interest Classification Scheme
