# Supplementary material for: Investigating where adolescents engage in moderate to vigorous physical activity and sedentary behaviour: An exploratory study
Source: PLoS One. 2022 Dec 6;17(12):e0276934. doi: 10.1371/journal.pone.0276934 (PMC9725162; doi:10.1371/journal.pone.0276934)
Supplement: S2 Table — (DOCX) [file pone.0276934.s003.docx]

**Table S2.** Participant characteristic by full sample and included participants

| **Characteristic** | **Full Sample**  **(n=69)** | **Included Participants**  **(n=34)** |
| --- | --- | --- |
| **Gender**  Female  Male | 45 (65.2)  24 (34.8) | 24 (70.6)  10 (29.4) |
| **Age^1^** | 16.1 ± 1.3 | 16.2 ± 1.2 |
| **Ethnicity**  White British  All other ethnic backgrounds | 55 (79.7)  14 (20.3) | 27 (79.4)  7 (20.6) |
| ***Area Level Deprivation***  1 (most deprived)  2  3  4  5 (least deprived) | 20 (29.0)  15 (21.7)  7 (10.1)  19 (27.5)  8 (11.6) | 11 (32.4)  7 (20.6)  4 (11.8)  7 (20.6)  5 (14.7) |
| **Self-drawn Neighbourhood Size** (km^2^) (n=53)^1^ | 2.7 ± 7.5 | 0.62 ± 1.1 |
| **MVPA Space Size** (km^2^)^1^ | 0.6 ± 0.5 | 0.62 ± 0.46 |
| **Sedentary Space Size** (km²)^1^ | 2.2 ± 2.3 | 2.2 ± 2.3 |
| Data are presented as n (%) unless stated otherwise.  ^1^Mean ± SD | | |
