# Supplementary material for: Investigating where adolescents engage in moderate to vigorous physical activity and sedentary behaviour: An exploratory study
Source: PLoS One. 2022 Dec 6;17(12):e0276934. doi: 10.1371/journal.pone.0276934 (PMC9725162; doi:10.1371/journal.pone.0276934)
Supplement: S1 File — (DOCX) [file pone.0276934.s004.docx]

Supplemental 1. Qualtrics Research Questionnaire

Q1 Thank you for participating in this study. Please take a few moments to answer the following questions. 


Please note that all of your information will be kept confidential and anonymous.

Q2 Name (please both first and last). This is only used to match post codes to data.

________________________________________________________________

Q3 Participant ID (please use the number provided for you)

________________________________________________________________

Q4 Email address (please use the one you will use to register for the app)

________________________________________________________________

Q5 Mobile Number *Optional-this will only be used for text reminders if you want them

________________________________________________________________

Q6 Age

________________________________________________________________

Q7 Post Code

________________________________________________________________

Q8 Gender

- Male (1)
- Female (2)
- Other (3)
- Prefer Not to Answer (4)

9 Ethnicity

- White (English/Welsh/Scottish/Northern Irish/British (1)
- White (Irish) (2)
- White (Gypsy or Irish Traveller) (3)
- Any other white background (4)
- Mixed/Multiple ethnic groups (5)
- Indian (6)
- Pakistani (7)
- Bangladeshi (8)
- Chinese (9)
- Any other Asian Background (10)
- African (11)
- Caribbean (12)
- Any other Black/African/Caribbean background (13)
- Any other ethnic group (14)
- Prefer not to answer (15)
